# Covalently Bonded Chitosan on Graphene Oxide via Redox Reaction

**DOI:** 10.3390/ma6030911

**Published:** 2013-03-07

**Authors:** Karina Bustos-Ramírez, Ana L. Martínez-Hernández, Gonzalo Martínez-Barrera, Miguel de Icaza, Víctor M. Castaño, Carlos Velasco-Santos

**Affiliations:** 1 Posgrado en Ciencia de Materiales, Laboratorio de Investigación y Desarrollo de Materiales Avanzados (LIDMA), Facultad de Química, Universidad Autónoma del Estado de México, Km.12 de la carretera Toluca-Atlacomulco, San Cayetano 50200, Mexico; E-Mails: ckl2833@hotmail.com (K.B.-R.); gonzomartinez02@yahoo.com.mx (G.M.-B.); 2 División de Estudios de Posgrado e Investigación, Instituto Tecnológico de Querétaro, Av. Tecnológico s/n Esq. M. Escobedo, Querétaro 76000, Mexico; E-Mail: almh72@gmail.com; 3 Centro de Física Aplicada y Tecnología Avanzada, Universidad Nacional Autónoma de México, AP 1-1010, Querétaro 76000, Mexico; E-Mails: deicazah@gmail.com (M.I.); meneses@unam.mx (V.M.C.)

**Keywords:** chitosan, grafting, graphene oxide, carbon nanostructure, redox reaction

## Abstract

Carbon nanostructures have played an important role in creating a new field of materials based on carbon. Chemical modification of carbon nanostructures through grafting has been a successful step to improve dispersion and compatibility in solvents, with biomolecules and polymers to form nanocomposites. In this sense carbohydrates such as chitosan are extremely valuable because their functional groups play an important role in diversifying the applications of carbon nanomaterials. This paper reports the covalent attachment of chitosan onto graphene oxide, taking advantage of this carbohydrate at the nanometric level. Grafting is an innovative route to modify properties of graphene, a two-dimensional nanometric arrangement, which is one of the most novel and promising nanostructures. Chitosan grafting was achieved by redox reaction using different temperature conditions that impact on the morphology and features of graphene oxide sheets. Transmission Electron Microscopy, Fourier Transform Infrared, Raman and Energy Dispersive spectroscopies were used to study the surface of chitosan-grafted-graphene oxide. Results show a successful modification indicated by the functional groups found in the grafted material. Dispersions of chitosan-grafted-graphene oxide samples in water and hexane revealed different behavior due to the chemical groups attached to the graphene oxide sheet.

## 1. Introduction

Chitosan, a polysaccharide composed of *β*-(1,4)-2-amino-2-deoxy-D-glucose, is the deacetylated product of chitin, *β*-(1,4)-2-acetamido-2-deoxy-D-glucose. Chitosan has been extensively investigated for several decades due to its biocompatibility, biodegradability, solubility in aqueous medium, presence of multiple functional groups and non-toxicity, which can be applied in molecular separation, food packaging film, artificial skin, bone substitutes, water treatment, electrochemical sensors and biosensors among others [1,2]. Also this biopolymer has been used to modify carbon nanostructures in order to improve dispersion stability in aqueous solution and biocompatibility [3,4,5]. Chemical modifications of carbon-based nanomaterials with biopolymers have been successfully achieved, opening a new way to a large number of opportunities, such as cell labeling, biosensors, bioactive molecular attachment and the preparation of nanocomposites with biocompatible and biodegradable polymers [2,6,7]. Specifically, chitosan has been used to modify carbon nanotubes by chemical grafting and non-covalent interactions [1,2,8].

On the other hand, today, graphene attracts as much attention as carbon nanotubes did in the last decade. So, this material, a single layer of carbon atoms in a closely packed honeycomb two-dimensional lattice, has engaged considerable interest from both the experimental and theoretical scientific communities [6,9,10,11]. Some of their most interesting characteristics are: Young’s modulus around 1.04 TPa [12], high thermal conductivity with an estimated value of 5300 W m^−1^ K^−1^, and consideration as a zero-bandgap semiconductor [13]. Each graphene sheet has a high aspect ratio, high modulus and high surface area [14]; graphene has been used in the development of theoretical and experimental sensing devices [15,16], in water remediation forming hybrid materials with magnetite [17] and in electronic devices [18,19]. Also, a large-scale pattern growth graphene film has been synthesized for produce electrodes [20,21].

One disadvantage of graphene is the characteristic poor dispersion observed also in other carbon based nanomaterials: nanotubes and fullerenes. Dispersion of carbon materials depends on different factors, such as polarity of the solvent, chemical group attached to carbonaceous structure, graphene sheets stack, nature of interactions between graphitic layers and the solvent, *etc*. Its importance is reflected in the great number of researches focused toward this aim, for instance: Stable dispersions of graphene sheets have been achieved using as dispersing agent amphiphilic polymers, alkylamines and hydrophilic carboxyl groups, among others [22]. Thus, functionalization of graphene has been considered as an important route for improving its dispersion [6,13,23,24,25]. Different authors have tried to overcome this disadvantage by producing graphene oxide, a graphite derivative with hydroxyl, epoxy and carboxyl groups covalently attached to its layers. This offers better dispersion in some solvents due to the functional groups joined to the graphene sheets [23,26]. For instance, graphene and graphite derivatives, functionalized with (1) polymers such as Poly-L-lysine [6], Poly(vinyl alcohol) [13] and (2) other molecules such as amine, aminoacids [23] or (3) charged with K ions via functionalization with strong base [26] have resulted in materials soluble in water. However, carbon sheet materials modified with other molecules such as alkyl chains yielded soluble materials in organic solvents namely chloroform, 1,2,4-trichlorobenzene [24] and tetrahydrofuran [25]. Therefore, the dispersion requirement for graphene oxide sheets has caused great interest focused on researching different routes towards their chemical modification. This is mainly for functionalization or chemical modification of the basal plane which is necessary to increase solubility and prevent the formation of π–π interactions, van der Waals interactions and scrolling [6,9]. Several kinds of organic molecules have been used in this sense, for instance amines [6,9,23], isocyanates [27], 1-pyrenebutyrate [22] and different polymers [13,28,29,30]. Recently chitosan was applied in different studies with graphene. For instance, as matrix in a bionanocomposite film for sensitive glucose sensing [31]; with tea polyphenol reduced graphene [32] and with keratin-grafted graphene oxide [33,34] for nontoxic and biocompatible biocomposites. In addition, true bonding interactions between graphene and chitosan (chemical or non-covalent) have also been seen recently. These studies include synthesis of water-soluble chitosan-grafted reduced graphene oxide [35]; magnetic chitosan grafted with graphene oxide [36]; covalent functionalization of graphene oxide with chitosan using different methods [37,38], and chitosan reinforced with graphene oxide to produce nanocomposites [39]. These researches combine the inherent characteristics of chitosan and the remarkable promise of graphene nanostructures. The carbohydrates, such as chitosan, provide a hydrophilic surface to the carbon nanostructures for covalent, absorptive or ionic linkages with bioactive molecules. Because of the interesting results that can be obtained, research on the attachment of carbohydrates to graphene oxide is in full development. Thus, this research presents grafting of chitosan onto a graphene oxide sheet achieved at different conditions of temperature, showing that this parameter is important for the final structure of graphene oxide grafted sheets. Morphology changes and different dispersion behavior in polar and non-polar solvents reflects the importance of chitosan in the graphene oxide sheet. It is known that stable dispersion and biocompatibility are features that can be generated in graphene oxide through non-covalent or grafting processes. In the case of grafting, this chemical modification generates interactions between functional groups of both chitosan and graphene oxide improving dispersion of this material in several solvents. Also this modification allows the possibility to progress in the incorporation of graphene oxide into diverse polymer matrices, including green and natural polymers; inasmuch as, graphene and graphene oxide have shown biocompatible behavior and low or non-cytotocixity, when are functionalized with some biomolecules [40,41,42,43]. This may attract more interest in the incorporation of chitosan in the permanent evolving area of nanotechnology.

## 2. Results and Discussion

Next the results obtained with the characterization of grafted graphene oxide with chitosan are presented together with the samples used as references. A summary of the nomenclature for all samples synthesized is shown in Table 1.

**Figure 1:**
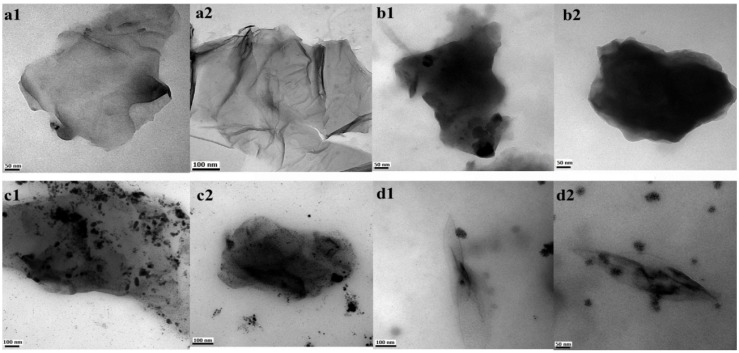
Transmission electron microscope (TEM) images of: (**a**) GEO, (**b**) CGEO1, (**c**) CGEO2 and (**d**) CGEO3 (see Table 1).

The graphene oxide sheet morphology (GEO) is observed in Figure 1a, illustrating its flakelike shape and the successful exfoliation of individual graphene oxide sheets at the nanoscale. This TEM micrograph shows the graphene oxide sheet as a thin extended film with a wrinkled surface. It has been reported that this wrinkled characteristic is an advantage, maintaining a high surface area to prevent collapse back into a graphitic structure [31]. TEM images of chitosan-grafted onto graphene oxide are shown in Figure 1b–d. As can be observed, temperature is an important factor that modifies grafting behavior, reflected in the morphology of CGEO1, CGEO2 and CGEO3 samples. The lower grafting temperature (55–60 °C for CGEO1) allows complete covering of chitosan onto the graphene oxide sheet (Figure 1b) preserving the film structure. However at 75–80 °C (CGEO2) the graphene oxide sheet is not fully covered by chitosan, and the biopolymer is observed only in some areas, in this case the plain layer structure is retained (Figure 1c). However, at 95–100 °C (CGEO3) chitosan grafting causes an important structural change, in which the chitosan-grafted graphene oxide sheet undergoes clear scrolling (Figure 1d). This effect can be attributed to the influence of hydroxyl and amino groups from chitosan, which can interact forming hydrogen bonding with hydroxyl, carbonyl and carboxyl moieties in the graphene oxide sheets. Thus, the morphology of grafted graphene could be correlated with the water quantity and hydrogen bonds depending on the temperature of grafting; due to, the loss of free water in chitosan occurring between 80 and 100 °C [44,45,46]. This indicates that better grafting conditions are found in CGEO1 because the reaction temperature is around 55–60 °C and more moisture is found. The other two samples change the morphology because less water is found in the material as the temperature is raised.

**Table 1:** Nomenclature of the carbon materials and nanomaterials synthesized.

Nomenclature	Description
GO	Graphite Oxide
GEO	Graphene Oxide
CGEO1	Chitosan grafted on graphene oxide at 55–60° C
CGEO2	Chitosan grafted on graphene oxide at 75–80° C
CGEO3	Chitosan grafted on graphene oxide at 95–100° C
CGEO	Chitosan grafted on graphene oxide

**Figure 2:**
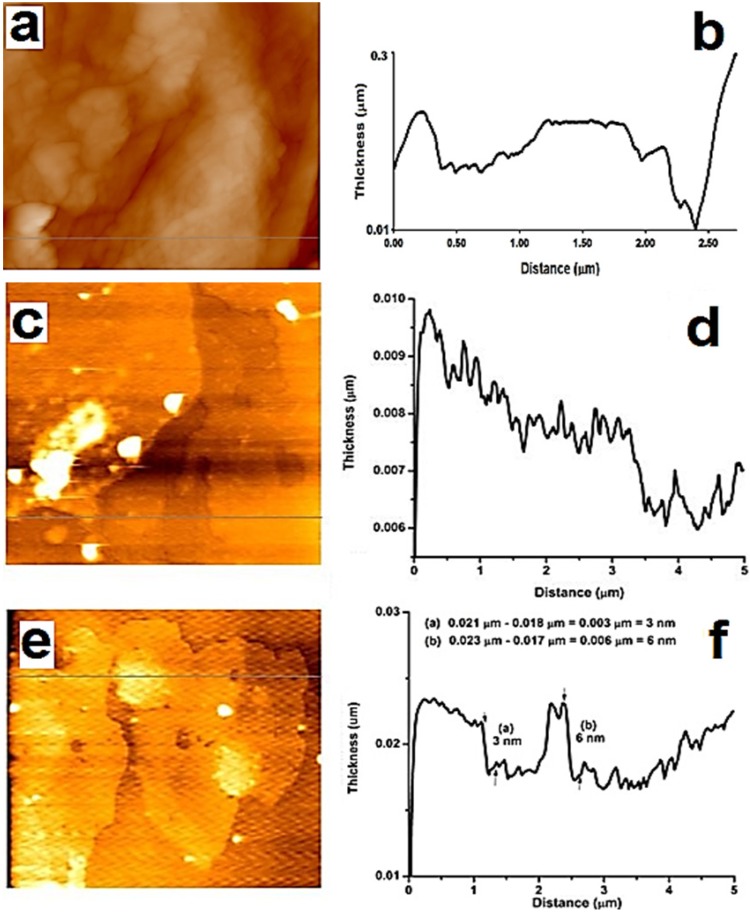
(**a**) Atomic force microscopy (AFM) image of chitosan acquired as is; (**b**) topography of chitosan; (**c**) AFM image of GEO; (**d**) topography of GEO; (**e**) AFM image of chitosan-grafted graphene oxide sheet and (**f**) topography of chitosan grafted graphene oxide sheet.

Figure 2a shows an atomic force microscopy (AFM) image of chitosan as obtained, where it is possible to observe an irregular structure with different thicknesses that reach up to 296 nm (topography graph Figure 2b). In contrast Figure 2e shows that grafted chitosan after reaction only covers a few nanometers when it is coupled to the graphene sheet.

Figure 2c,d show AFM images of an irregular surface of graphene oxide sheets, with notched shapes and roughness, the average height was measured at 0.56 nm. The functionalization in the basal plane (verified by Fourier transform infrared spectroscopy (FTIR)) and the crumpling of graphene sheets (appreciated in TEM images) could cause roughness, as reported by Worsley *et al.* [25]. AFM images of chitosan-grafted graphene sheets are shown in Figure 2e,f, where it is possible to observe variations in height from 3 to 6 nm, this increment in thickness is due to polysaccharide grafting covering both sides of GEO. In Figure 2e dense areas are observed, corresponding to a height of 6 nm, these are attributed to bundles of chitosan chains grafted onto GEO sheets.

**Figure 3:**
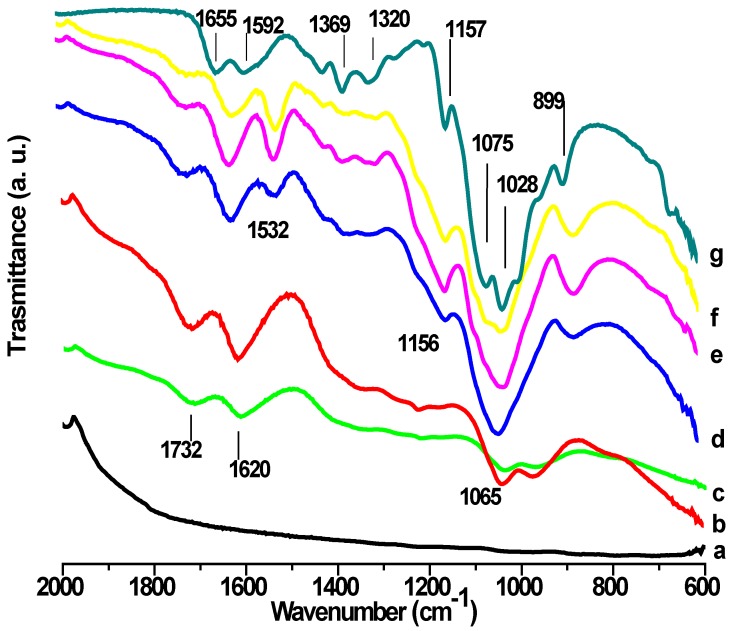
Fourier transform infrared spectroscopy (FTIR) spectra of: (**a**) graphite; (**b**) GO; (**c**) GEO; (**d**–**f**) chitosan-grafted graphene oxide at different temperatures of treatment: (**d**) CGEO1; (**e**) CGEO2; (**f**) CGEO3 and (**g**) chitosan.

Different functional groups were observed in all graphene samples through FTIR analysis (Figure 3). Pure graphite is infrared inactive as is observed in Figure 3a, whereas GEO and GO spectra (Figure 3b,c respectively) show several distinctive signals such as: 1732 cm^−1^, v(C=O) of carboxyl groups; 1620 cm^−1^, v(C=C); and 1065 cm^−1^, v(C–O). These signals provide evidence of the presence of oxygen-containing groups, caused by the chemical reaction. These moieties have also been reported by different authors [6,9,27].

The effect of chemical modification due to grafting chitosan onto graphene oxide is observed in CGEO1, CGEO2 and CGEO3 spectra (Figure 3d–f respectively). New signals are observed because of interactions between carbonyl and hydroxyl groups from GEO and chitosan moieties: amino, hydroxyl and RNHCOCH_3_, this last from remaining acetylated parts of chitosan. At 1535 cm^−1^ a new band is observed in CGEO spectra (Figure 3d–f). It can be assigned to the combination of the v(C–N) with the δ(CHN) (amide II) [47], which corresponds to amides or carbamate esters formed during the grafting reaction [29]. The signal at 1156 cm^−1^ is attributed to v_a_(C–O–C) and the band at 899 cm^−1^ corresponds also to C–O–C, in both cases related to the glycosidic linkage [47]. These signals, typical in chitosan, were absent in GEO and GO spectra, but they appear with a clear shift (888 cm^−1^) in CGEO spectra. This indicates that C–O–C bond vibrations are also modified due to grafting treatment; when C–O– bonds of chitosan interact with GEO in CGEO hybrids. Thus, not only amine groups can take part in grafting, also the OH, of the chitosan structure can play an important role.

**Figure 4:**
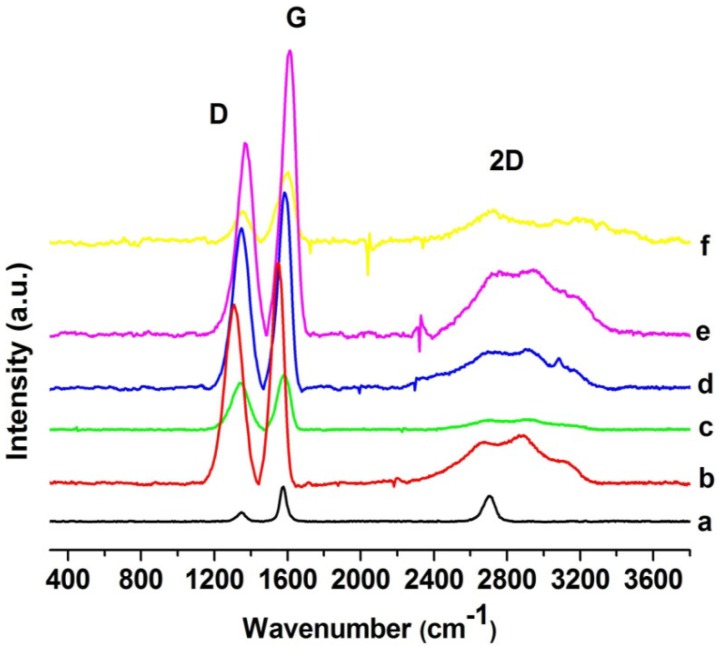
Raman spectra of: (**a**) graphite; (**b**) GO; (**c**) GEO; (**d**–**f**) chitosan-grafted graphene oxide at different temperatures: (**d**) CGEO1; (**e**) CGEO2; (**f**) CGEO3.

Raman spectra of carbon allotropes, such as graphite, show distinctive signals: A strong band close to 1580 cm^−1^ (G band), a line around 1350 cm^−1^ (D band) and 2D band in the region of 2700 cm^−1^ [10,25,26,47,48,49,50]. The tangential G mode corresponds to the first-order scattering of the E2g mode in-phase vibration of the graphite lattice [25,48,49,50]. D band is due to the out-of-plane breathing mode of the sp^2^ atoms, it reveals the presence of certain defects, whereas 2D is the second order of this vibrational mode [48,49,50,51,52,53]. In the spectrum of pure graphite (Figure 4a) it is observed that D and G modes are weak, but after oxidation (GO, Figure 4b) and exfoliation (GEO Figure 4c) both modes become wider and increase their intensity. The well defined peak of the 2D band in the pure graphite spectrum (Figure 4a) undergoes certain changes in graphene spectra: It goes out of shape in GO (Figure 4b) and even disappears in GEO (Figure 4c). These variations confirm the modification and layer separation of the carbon structure, since these Raman results are in agreement with reports from different authors, which have established that structural defects, crystallinity, doping, layer numbers and ordering of the sp^2^ sites affect positions, intensities and widths of the D and G bands [11,25]. In the same way, the intensity and breadth of the 2D band in graphene depends on the number of layers. The 2D band is a second-order process related to a phonon near the K point in graphene, activated by double resonance processes, which are responsible for its dispersive nature and cause a strong dependence on any perturbation of the electronic and/or phonon structure of graphene. Thus, the 2D band is very predisposed to characterize specific sp^2^ nanocarbons [54,55,56]. Consequently, the changes of the 2D band in graphene oxide that are shown in Figure 4 are related to considerable defects in the graphene sheet, produced by oxidation [51].

Similar behavior is observed for chitosan-grafted graphene samples (CGEO1 Figure 4d, CGEO2 Figure 4e and CGEO3 Figure 4f), D and G bands show a shift, broadening and increasing as a result of the covalent attachment of the polysaccharide to the graphene sheet. A comparable result was published by Chattopadhyay *et al.* [24]. However the most evident changes in Raman spectra of the grafted samples are observed in the 2D band. As previously mentioned this band is very sensitive to changes in the graphene sheets [54,55,56] and its broadening is evidently related to the changes produced in the graphene oxide layers by the grafted chitosan.

Chemical modification is also revealed by the presence of oxygen and nitrogen in GEO and grafted samples, which was verified by Energy dispersive X-ray spectroscopy (EDS). Table 2 shows a maximum percentage of carbon in GEO (60.45%) accompanied by 38.59% of oxygen related to hydroxyl and carbonyl groups as was demonstrated by FTIR results. Grafting treatment diminishes the content of carbon to around 23%–26%, but oxygen is dramatically increased (71%–72%) and nitrogen is also detected. The latter come from the amine groups of chitosan. In spite of washing, all samples present a residual content of sulfur due to H_2_SO_4_ used in the redox reaction. Also, chitosan analysis obtained by Salam *et al.* of similar samples to that used in this research is added in the table as reference for the nitrogen, carbon and oxygen content of this natural polymer [57]. It is important to mention that we attributed the low content of carbon and high content of oxygen in all grafted samples due to EDS realized on the surface of the grafting material, and in this superficial part seems to prevail in oxygen groups instead of carbon. Thus, in spite of, a higher carbon content which should be found in grafted samples, due to grafting achieved on the surface; oxygen predominates as dangling bonds, of both, graphene oxide and grafted chitosan. Also, we suppose the influence of moisture due to the presence of chitosan could influence these obtained values in the grafted samples due to the hydrophilic nature of chitosan.

**Table 2:** Results of energy dispersive spectra (EDS) of graphene oxide, chitosan-grafted graphene oxide at 55–60 °C (CGEO1), 75–80 °C (CGEO2) and 95–100 °C (CGEO3).

**Element**	**Graphene Oxide** (Weight %)	**CGEO1** (Weight %)	**CGEO2** (Weight %)	**CGEO3** (Weight %)
	C	60.45	26.06	23.63	24.32
O	38.59	72.07	71.69	71.54
N	0	0.12	1.6	2.95
S	0.97	1.75	2.73	0.78

Note: Chitosan EDS analysis: C 40.18%, O 45.24%, N 7.24% [57].

**Figure 5:**
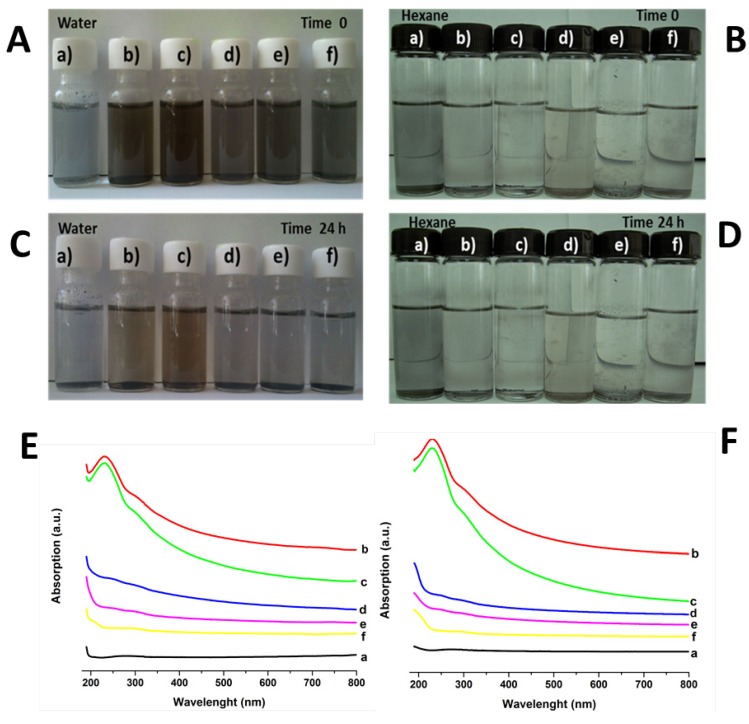
Dispersion study of: (**A**) water after sonication; (**B**) hexane after sonication; (**C**) water after sonication and 24 h kept at rest; (**D**) hexane after sonication and 24 h kept at rest; (**E**) UV-Vis spectra of samples dispersed in water after sonication; (**F**) UV-Vis spectra of samples dispersed in water after sonication and 24 h kept at rest: (**a**) graphite; (**b**) GO; (**c**) GEO; (**d**–**f**) chitosan-grafted graphene oxide at: (**d**) CGEO1; (**e**) CGEO2; (**f**) CGEO3.

Finally, our dispersion study provides results that reflect a strong affinity between water and the different groups attached to graphene sheets (Figure 5A). Dispersions with water show the darkest color for the longer time. Pure graphite can be dispersed only partially and a high number of particles remain adhered to the container walls. However, the presence of carboxyl and hydroxyl groups in GO and GEO (columns b and c in Figure 5C) allows the formation of a stable dispersion in water even after 24 h. For chitosan-grafted samples (columns d, e and f in Figure 5C), the hydrophilic nature of this polysaccharide facilitates the interactions between both water and amine, as well as carbonyl and hydroxyl groups. However, it is important to mention that dispersion related to graphene oxide grafted with chitosan obtained at the temperature range of 55–60 °C (CGEO1-column d, Figure 5C.) is better after 24 h than the other samples of grafted graphene oxide. CGEO-1 is also the sample where chitosan covers the graphene oxide sheets in a better way (Figure 1b). On the other hand, dispersion of graphene oxide in hexane is poor (Figure 5B,D), the absence of suitable chemical groups to interact and the non polar nature of the solvent cause the nanosheets to precipitate rapidly, regardless of moieties attached to GO, GEO and grafted samples. For the samples dispersed in water with the aim to make clear the differences in dispersion between the grafted samples (CGEO1, CGEO2 and CGEO3) there is included in Figure 5E, F the UV-Vis spectra of all samples. In both figures after sonication (Figure 5E) and after retention for 24 h at rest (Figure 5F) the spectra, show similar dispersion behavior. The peak related to the π–π* transitions near to 230 nm and the shoulder at 300 nm due to n–π* transition [37] are clear and more intensive in GO and GEO spectra, giving evidence that better dispersion is reached even maintaining at rest for 24 h. Also, it is evident than CGEO1 is the grafted sample that maintains absorption due to better dispersion than the other two grafted samples (CGEO2 and CGEO3). This corroborated that all grafted samples have different dispersion features even after maintaining at rest for 24 h.

## 3. Experimental Section

### 3.1. Materials

Chitosan (CS) (degree of deacetylation >85%) was purchased from Sigma-Aldrich. Graphite rods, spectro-grade (ash content <2 ppm) were obtained from Electron Microscopy Science. Potassium permanganate (KMnO_4_) was acquired from Merck. Sulfuric acid (H_2_SO_4_) (98%), hydrogen peroxide (H_2_O_2_) (30%), acetone (99%) and hexane (99%) were purchased from Baker and malic acid (99%) was supplied by Fluka.

### 3.2. Graphene Oxide Sheet Preparation

Graphite oxide (GO) was obtained from graphite by a modified Hummers method [26], only quantities of reagents were varied. H_2_SO_4_ (46 mL) was added into the reaction flask maintaining 0 °C (±2°) with an ice bath, graphite (2 g) and KMnO_4_ (6 g) were added slowly, then the temperature was increased to 35 °C (±2°) and the mixture was stirred with a magnetic stirring bar for 2 h. Later, excess water was incorporated and H_2_O_2_ (10 mL) was added until there was no gas production. A glass filter was used to wash with water and a brown powder was obtained (GO), which was dried at 65 °C for 12 h. Then, a solution containing 100 mg of dried GO in 10 mL of H_2_O was produced, this solution was sonicated for 3 h at room temperature with the aid of an ultrasonic bath (Branson 1510R-MTH with frequency of 50–60 Hz), thus producing graphene oxide sheet solution (GEO), which was conserved for its subsequent characterization.

### 3.3. Chitosan Grafting on Graphene Oxide

**Figure 6:**
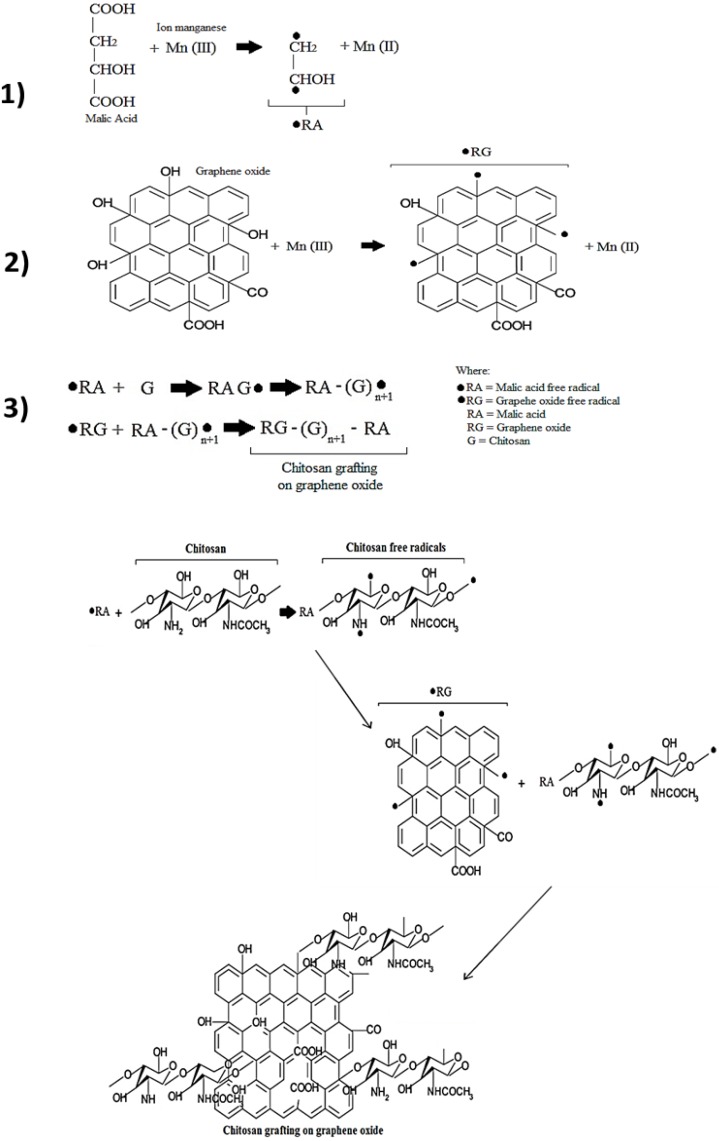
Reaction scheme of chitosan grafting onto graphene oxide.

The solution obtained in the last procedure was constantly stirred in a reaction flask whereby 125 mg of chitosan, 37.5 mg of KMnO_4_, 50 mg of malic acid and 0.75 mL of H_2_SO_4_, were added. The mixture reacts for 3 h at fixed temperature. In order to observe the temperature effects on chitosan graftings, three ranges were applied: 55 to 60 °C, 75 to 80 °C and 95 to 100 °C (CGEO1, CGEO2 and CGEO3 respectively). Finally the reaction products were washed with acetone and dried for 24 h at room temperature. A scheme of this reaction taking as basis previous grafting works [58,59] is shown in Figure 6. In this scheme the function of each reactant in the grafting reaction is illustrated.

### 3.4. Analytical Characterization

Transmission Electron Microscopy (TEM) micrographs were obtained using a JEOL TEM-1010 microscope operating at 80 kV. The AFM images were recorded using an Atomic Force Microscope, AA5000 Multifunction SPM Systems. In the case of chitosan samples, the scans were performed in contact mode, operating at a frequency of 5 kHz. For graphene oxide and grafted samples AFM Digital Instrument CP-II was used, the scans were performed in non-contact mode, operating at a frequency of 200 kHz and the scanning speed was 0.5 Hz. Micro Raman (Dilor, Lab Ram) measurements were carried out at 368 nm wavelength incident laser light with a spectral resolution of 1 cm^−1^. Fourier transform infrared spectroscopy (FTIR) was recorded in a FT-IR Avatar Model 360 by Nicolet spectrometer, at a resolution of 4 cm^−1^ and by the attenuated total reflectance technique (ATR). Finally energy dispersive X-ray spectroscopy (EDS) was done in a JEOL JSM-6060LV SEM using an Oxford Inca X-sight system. All samples for EDS were prepared previously as solid disks, which were fastened to a double-sided adhesive tape attached to aluminum stubs.

### 3.5. Dispersion Study

Dispersion measurements were performed maintaining the carbon material-solvent relation as 2:10, the same as proposed by Chattopadhyay *et al.* [24]. Graphite and GO (2 mg) were dispersed in 10 mL of solvent, and sonicated for only 15 min in order to preserve their characteristic layers. In turn GEO, CGEO1, CGEO2 and CGEO3 (2 mg) were dispersed also in 10 mL of solvent and sonicated for 15 min. For this study the solvents were: water and hexane (in order to observe the behavior in solvents with opposite polarity). All dispersions were left to settle for 24 h at room temperature.

To complement the dispersion study UV-Vis spectra were obtained in UV Vis DR 5000 HACH equipment in scan mode of 190 nm to 800 nm for the samples dispersed in water (after sonication and 24 h after settling), only water dispersions were run, inasmuch as, dispersions in hexane were very poor.

## 4. Conclusions

Results show successful grafting indicated by the functional groups of graphene oxide and chitosan moieties, since carboxyl, carbonyl, and hydroxyl groups are found in GO, GEO and grafted samples. The presence of amide bands in FTIR of grafted samples is significant as this reveals the chemical interaction between the amine group from the polysaccharide and the oxygen-containing groups of GEO. The TEM and AFM images demonstrate important changes in the morphology of GEO sheets in comparison to the chitosan-grafted samples. Also grafted conditions produce important changes in the grafted graphene oxide structure as was shown by Raman spectroscopy, where G, D and 2D bands present important shifts related to the linked chitosan. These changes in the graphene structure are also corroborated by microscopy images. In spite of morphologic variations dispersion in water is not affected significantly as hydroxyl and carbonyl groups remain attached to the graphene sheets. For all samples the most suitable solvent was water, since polarity and hydrogen bonds play an important role in dispersion behavior. The temperature range used in the grafting reactions plays an important role in the morphology of grafted sheets and therefore for solubility properties. Thus, chitosan grafting through redox reaction is an appropriate route to improve dispersion and provides an interesting umbrella for bio-related applications, such as nanocomposites, biocomposites, biosensors, biomedical materials, *etc*., taking advantage of the potential associated with this nanoscale material
